# An Unusual and Complicated Course of a Giant Cell Tumor of the Capitate Bone

**DOI:** 10.1155/2016/3705808

**Published:** 2016-10-25

**Authors:** Ingo Schmidt

**Affiliations:** SRH Poliklinik Gera GmbH, Straße des Friedens 122, 07548 Gera, Germany

## Abstract

A 51-year-old female patient presented with a carpal giant cell tumor (GCT) of the right capitate bone. The lesion was initially misdiagnosed as having an osteomyelitis. First, the diagnosis of a benign GCT was confirmed by histological examination. Second, an intralesional curettage and packing of the cavity with cancellous iliac crest bone grafts combined with a fusion of the third carpometacarpal (CMC III) joint were carried out. Third, due to a secondary midcarpal osteoarthritis and a secondary scaphoid nonunion, the CMC III joint fusion plate was removed and the midcarpal joint completely excised. Fourth, in the absence of recurrence of GCT, a four-corner fusion (4CF) with a corticocancellous iliac crest bone graft and complete excision of the scaphoid bone had to be performed. Fifth, a total wrist arthroplasty (TWA) was performed due to hardware failure of 4CF with migration of a headless compression screw into radiocarpal joint which led to erosion of articular surface of the distal radius. At the 3-year follow-up that includes a 1-year follow-up after TWA, there was no recurrence of GCT, and the TWA was not failed. The patient reported that she would have the motion-preserving TWA again.

## 1. Introduction

Giant cell tumor (GCT) of the bone is a rare, benign, and locally aggressive tumor, constituting 4-5% of all primary bone tumors and 18–20% of all benign bone tumors. Two malignant variants are known—first, the primary malignant GCT that was found in up to 10% of cases; and second, the rare sarcomatous proliferation appears inside the lesion previously documented as a benign GCT. Usually, GCT occurs in skeletally mature individuals, the patient's age ranges from 20 to 50 years, and the peak age incidence is in the third and fourth decade of life with slight female predominance. Typically, the tumor site is subchondral at the long bone metaepiphysis, especially the distal radius and femur, and proximal humerus and tibia [1–3].

GCT of the carpal bones is a very rare entity, only few case reports have described unifocal or multifocal appearance involving the proximal and/or distal row [4, 5]. We present an unusual and complicated course of GCT of the capitate bone which required five surgical procedures within two years. To our knowledge, such a case finally resulting in a total wrist arthroplasty (TWA) has not been reported thus far.

## 2. Case Report

**Figure 1:**
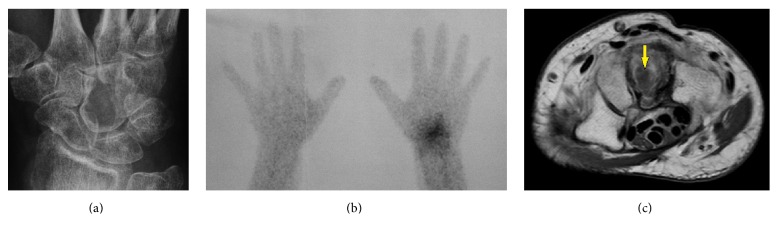
Case report (preoperative diagnostic findings): (a) PA radiograph showing lytic lesion in capitate bone with cortical thinning and cortical destruction distally; (b) bone-granulocyte scintigraphy of both hands showing increased radionuclide uptake in the region of right carpal bones 45 minutes after application; (c) axial MRI sequence showing low to intermediate signal intensity in capitate bone and no soft-tissue extension* (arrow)*.

The posteroanterior (PA) radiograph of a 51-year-old female patient showed an intraosseous lytic lesion of the right capitate bone (Figure 1(a)) that was associated with a marked radionuclide uptake in all carpal bones using bone-granulocyte scintigraphy (Figure 1(b)) and magnetic resonance imaging (MRI) demonstrated low to intermediate signal intensity of the lesion without soft-tissue extension (Figure 1(c)). Due to these findings, an osteomyelitis of the capitate bone was suggested by the radiologist. At first, free intraosseous sample was taken that revealed a benign GCT in histological examination. The chest radiograph did not show pulmonal pathology. Second, intralesional curettage and packing of the cavity with cancellous iliac crest bone grafts combined with a fusion of the third carpometacarpal (CMC III) joint using a 2,0 mm titanium plate (Medartis, Basel, Switzerland) were performed.

**Figure 2:**
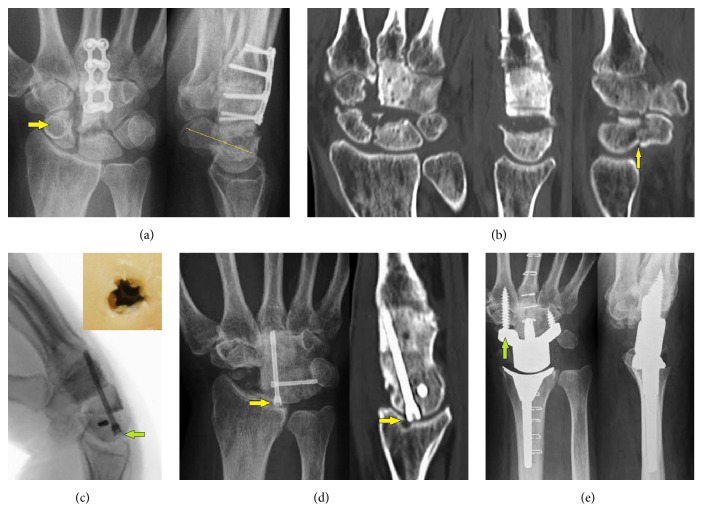
Case report (course): (a) PA and lateral radiographs showing union of CMC III joint fusion, severe destruction of midcarpal joint, positive ring sign* (arrow)*, and distinctive volar tilt of capitate bone* (line)*; (b) coronar and sagittal CT scans showing removal of CMC III joint fusion plate, solid bony structure of reconstructed capitate bone, no recurrence of GCT, complete union of CMC III joint fusion, complete resection of midcarpal joint for histological examination, and humpback deformity due to a nonunion at the waist of scaphoid bone* (arrow)*; (c) intraoperative clinical photograph and lateral fluoroscopy demonstrating safe subchondral placement of the longitudinally inserted 4CF headless compression screw at the lunate bone* (arrow)*; (d) PA radiograph and sagittal CT scan showing loosening and migration of longitudinally inserted compression screw into radiocarpal joint with erosion of articular surface at the distal radius* (arrows)*; (e) postoperative AP and lateral radiographs demonstrating correct alignment of TWA, note the scaphoid augment of carpal component* (arrow)*.

One year after, the PA and lateral radiographs showed union of the CMC III joint fusion without hardware failure, but there was pronounced destruction at the proximal pole of the reconstructed capitate bone and at the distal facet of lunate bone, and a positive ring sign associated with distinctive volar tilt of scaphoid bone in the absence of an injury after the second procedure (Figure 2(a)). Third, the CMC III joint fusion plate was removed and the midcarpal joint completely excised. The histological examination did not show recurrence of GCT but revealed pronounced midcarpal osteoarthritis (OA). Intraoperatively, a nonunion at the waist of scaphoid bone was seen. Postoperatively, computed tomography (CT) scans showed complete union of CMC III joint fusion associated with sufficient osseointegration of cancellous bone grafts at the capitate bone and additionally the humpback deformity due to a nonunion at the waist of scaphoid bone (Figure 2(b)). Fourth, an open 4CF with excision of the entire scaphoid bone using a corticocancellous iliac crest bone graft and two cannulated headless titanium compression screws (Medartis, Basel, Switzerland) was performed. One 2,2 mm screw was transversely inserted into triquetrum bone, and the second 3.0 mm screw was longitudinally inserted into the reconstructed capitate bone in an antegrade manner breaching the articular surface of lunate bone. Intraoperatively, there was a safe subchondral placement of the longitudinally inserted compression screw at the lunate bone (Figure 2(c)).

One year after 4CF, a migration of the longitudinally inserted compression screw into radiocarpal joint with erosion of the articular surface at the distal radius was present (Figure 2(d)). The patient declined a total wrist fusion (TWF), and a TWA using the relatively new angle-stable Maestro™ Wrist Reconstructive System (WRS, Biomet, Warsaw, Indiana/USA) with the use of a scaphoid augment distally was performed (Figure 2(e)). After that, the course was uncomplicated.

**Figure 3:**
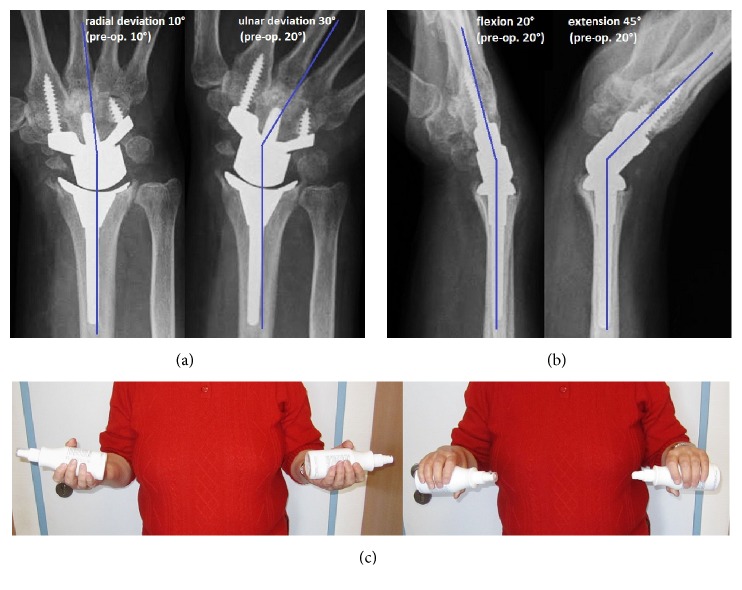
Case report (3-year follow-up): (a) PA radiographs showing no recurrence of GCT, unchanged correct alignment of TWA without any signs of loosening, and no impingement with terminal ranges of motion; (b) lateral PA radiographs with terminal ranges of motion showing no instability of TWA; (c) clinical photographs demonstrating 90° supination and 90° pronation of both forearms.

At the 3-year follow-up (including 1-year follow-up after TWA), the radiographs did not show recurrence of GCT, the TWA was not loosened, and there were no signs of impingement nor instability with terminal ranges of motion (Figures 3(a)-3(b)). Pain improved from 8 to 2 in visual analog score (0–10 points). Patient-rated wrist evaluation (0–100 points) improved from 88 to 37. Wrist extension and wrist ulnar deviation improved from 20° to 45° and from 20° to 30°. Wrist flexion and wrist radial deviation were equal to preoperative. The forearm motion arc with 90° supination and 90° pronation was 100% to the opposite forearm (Figure 3(c)). The patient reported that she would have the motion-preserving TWA again.

## 3. Discussion

GCTs of bone rarely occur in the hand. When they do, the metacarpals and phalanges are the most commonly affected bones. Based on a review of 1228 cases of GCT of all bones, Averill et al. [5] found that only 3% of cases occurred in the hand and extremely rare with 0,32% in carpal bones, patients are often younger than patients with appearance in other locations, recurrence is more rapidly than in other locations, the incidence of multifocal appearance of GCT of bones in the hand is 18%, and 87% of GCTs in the hand treated by curettage recurred. GCT of carpal bones affected in 31% the hamate, in 24% the capitate, in 14% the scaphoid, in 10% the lunate, in 7% the triquetrum, in 7% the trapezium, in 7% the trapezoid, and multifocal appearance was found in 14% of all cases [4]. Multifocal GCT of carpal bones and multicentric occurrence involving nine sites of both upper extremities including the lunate bone in a course over 16 years were also described in two skeletally immature male patients with age of 14 and starting with age of 13 years [6, 7].

In a review of literature including hand published articles from 1935 to 2005, only 29 cases of GCT of carpal bones (averaged age of patients 32,6 years ranging from 16 to 80 years, data from three publications not available) could be found by Shigematsu et al. [4], and the high incidence of local recurrence with 24% of all cases was associated only with performed intralesional procedures and recurrence occurred between three months and four years, whereas in cases treated with an excisional procedure recurrence did not occur. Why the marked midcarpal OA and humpback deformity of the scaphoid due to a nonunion after the secondary procedure (curettage/cancellous bone grafting/CMC III joint fusion) occurred in our case is unclear.

Radiographically, GCT of carpal bones resembles other lytic lesions. As with other musculoskeletal neoplasm, CT and MRI are superior to conventional radiographs [8]. On the other hand, benign GCT of bone can also initially misdiagnosed on MRI as a malignancy [9]. Bone scintigraphy demonstrates increased radionuclide uptake in the vast majority of GCTs; however, bone scintigraphy is nonspecific, does not aid in the detection of GCT, does not differentiate benign from malignant GCT, and is most likely secondary to other bony abnormalities or local and/or regional hyperemia [10]. For detection of benign GCT of bone, histological examination of free intralesional samples is absolutely necessary.

Treatment strategy for GCT of bone has been evolved over the years with different surgical options, which can be summarized under two main categories: first, intralesional curettage and/or chemical treatment (hydrogen peroxide, phenol, and alcohol) and/or cryosurgery (instillation of liquid nitrogen), followed by packing of the cavity with bone grafts, bone graft substitutes, and/or polymethylmethacrylate (PMMA); and, second, en bloc resection of the entire tumor followed by reconstruction in the form of arthroplasty or arthrodesis using nonvascularized or vascularized bone grafts. When using an intralesional procedure, meticulous high-speed burring is recommended to improve the quality of curettage, but the use of autologous bone grafts is likely unable to prevent recurrence sufficiently [11, 12]. In contrast to autologous bone grafting, it has been reported that combined treatment of selective GCT in the hand with curettage, cryosurgery, and additional cementation (PMMA) appears to be safe and effective [13]. In a review of literature, it has been reported that some types of GCT of bones still have the ability to metastasize in the presence of a recurrence rate up to 45% [14]. Approximately, up to 3% of GCTs metastasizes to lung at certain time points after the confirmed diagnosis [15], and for patients who experienced local recurrence it has been estimated that they can have a six-fold higher risk of lung metastasis [16].

For treatment of GCT of carpal bones, care must be taken when using an intralesional curettage with or without bone grafting, and an excisional procedure is recommended to prevent recurrence [17–19]. If the scaphoid, lunate, or triquetrum is affected, proximal row carpectomy (PRC) or complete excision of the involved bone combined with an intercarpal fusion is the method of choice [20–22]. The use of retrograde or, such as in our case, antegrade inserted headless compression screws for 4CF has proven to be a suitable and reliable option with an union rate up to 94% [23–26], but hardware migration into radiocarpal joint despite complete union of 4CF, such as in our case, is a concern. Richards et al. [25] reported on proximal backing out of the screws in 14% of 21 treated patients; and it was also observed in all of four treated patients who underwent the insertion of headless compression screws in antegrade manner after averaged six months (4–8 months) despite union of all 4CFs [27]. If the distal row is affected, complete excision of the involved bone combined with an intercarpal fusion is the method of choice as well [28]; however, complete excision of the capitate without reconstruction of distal row or distal row carpectomy also can be surgical option [18, 29]. Vergara-Fernández et al. [30] reported on complex replacement of excised carpal bones using a long corticocancellous bone graft from the third metacarpal bone which was distracted by an external fixateur in a 15-year-old male patient. Averill et al. [5] reported on one case with multifocal carpal, metacarpal, and phalangeal appearance in an 18-year-old male patient that required a ray amputation. For recurrence of GCT after a primary excisional procedure or failed intercarpal fusion, total wrist fusion (TWF) with the use of large corticocancellous iliac crest bone grafts is to be considered as one salvage option. The overall complication rate of 4CF is 29% versus 14% for PRC, respectively [31]; main complications of 4CF are delayed or nonunion and/or, such as in our case, hardware failure [32], and the reported conversion rate to a TWF after a failed 4CF or PRC (including conversion to TWA) ranges from 8,7 to 14,8% [33–36].

**Figure 4:**
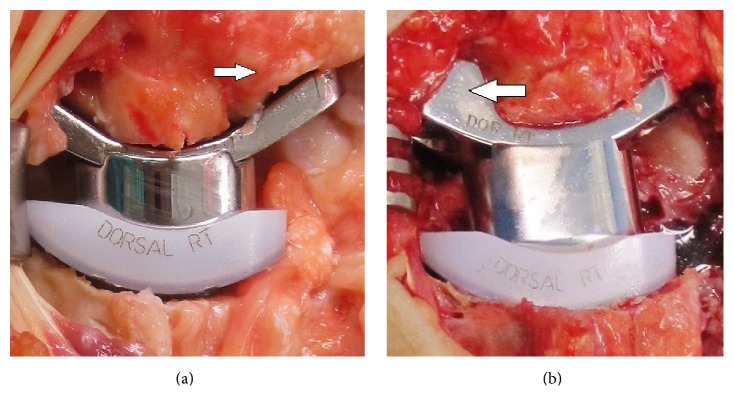
Technical note (Maestro WRS): (a) Clinical photograph demonstrating the use of carpal component without scaphoid augment, the distal pole of scaphoid bone is not excised* (arrow)*; (b) clinical photograph demonstrating the use of carpal component with scaphoid augment* (arrow)*, the entire scaphoid bone is excised.

TWA is the motion-preserving alternative to TWF, and it has proven to be successful after failed 4CF or PRC in single cases [35–40]. For this purpose in our case, the essential prerequisite was that the bone stock of the primary reconstructed capitate bone demonstrated a solid bony structure for safe fixation of the carpal TWA monoblock component with its central peg into the reconstructed capitate in the absence of local recurrence of GCT or local postoperative osteitis. The Maestro total wrist, developed by* Strickland/Palmer/Graham* in 2002 and available since January 2005, is a biaxial-anatomical third-generation type that is current in use, and first encouraging short-term results were published in 2009 [41]. In a single-center study, published in 2015, the cumulative implant survival after eight years (*N* = 68) is reported to be 95%, and at the 5-year follow-up radiographic loosening was present only in 2% of all cases [42]. Currently, the Maestro total wrist achieves the most favorable functional outcome as compared to other third-generation types (Remotion, Universal 2), and it may be justified in preserving resection-related carpal height due to its three various carpal heads in combination with its design of ellipsoid surface articulation [43]. Such as in our case, the design of implant allows the excision of the entire scaphoid bone accompanied with its replacement utilizing a carpal component that incorporates various scaphoid augments; therefore, it is not always necessary to attempt fusion of the distal pole of the scaphoid to the surrounding carpal bones ([41], Figures 4(a)-4(b)).

However, patients undergoing treatment with TWA must be prepared that it might end with TWF; therefore, limited bone resection is a common feature of all contemporary wrist replacements [44, 45]. The Maestro total wrist has demonstrated uncomplicated conversion to TWF [40, 42, 43, 46].
